# Aurora kinase A revives dormant laryngeal squamous cell carcinoma cells via FAK/PI3K/Akt pathway activation

**DOI:** 10.18632/oncotarget.10233

**Published:** 2016-06-23

**Authors:** Li-yun Yang, Chang-yu He, Xue-hua Chen, Li-ping Su, Bing-ya Liu, Hao Zhang

**Affiliations:** ^1^ Department of Otolaryngology, Ruijin Hospital, School of Medicine, Shanghai Jiaotong University, Shanghai, China; ^2^ Shanghai Key Laboratory of Gastric Neoplasms, Shanghai Institute of Digestive Surgery, School of Medicine, Shanghai Jiaotong University, Shanghai, China

**Keywords:** laryngeal cancer, aurora kinase A, FAK, PI3K, Akt

## Abstract

Revival of dormant tumor cells may be an important tumor metastasis mechanism. We hypothesized that aurora kinase A (AURKA), a cell cycle control kinase, promotes the transition of laryngeal squamous cell carcinoma (LSCC) cells from G0 phase to active division. We therefore investigated whether AURKA could revive dormant tumor cells to promote metastasis. Western blotting revealed that AURKA expression was persistently low in dormant laryngeal cancer Hep2 (D-Hep2) cells and high in non-dormant (T-Hep2) cells. Decreasing AURKA expression in T-Hep2 cells induced dormancy and reduced FAK/PI3K/Akt pathway activity. Increasing AURKA expression in D-Hep2 cells increased FAK/PI3K/Akt pathway activity and enhanced cellular proliferation, migration, invasion and metastasis. In addition, FAK/PI3K/Akt pathway inhibition caused dormancy-like behavior and reduced cellular mobility, migration and invasion. We conclude that AURKA may revive dormant tumor cells via FAK/PI3K/Akt pathway activation, thereby promoting migration and invasion in laryngeal cancer. AURKA/FAK/PI3K/Akt inhibitors may thus represent potential targets for clinical LSCC treatment.

## INTRODUCTION

Laryngeal squamous cell carcinoma (LSCC) is one of the most common head and neck squamous cell carcinomas (HNSCC), and arises from the larynx epithelium with high metastasis rates and poor prognosis [1, 2]. Currently LSCC patient therapies include chemotherapy, radiotherapy and surgery, alone or in combination [3]. While treatment prolongs patient survival, metastatic tumor growth severely reduces overall survival rates [4, 5]. Elucidating the mechanisms of LSCC metastasis will be essential for identifying potential molecular targets to improve patient survival and quality of life.

Years after initial treatment, LSCC patients may develop local remnant or disseminated tumors. This phenomenon can be explained by tumor dormancy, a stage in tumor progression in which residual disease is present, but asymptomatic [6]. Revival of dormant tumor cells may be important in tumor recurrence and metastasis in cancer of the lung [7], breast [8] and prostate [9], as well as HNSCC [10, 11]. However, mechanisms of tumor dormancy regulation are still largely unclear.

Aurora kinase A (AURKA), an oncogene [12], controls the cell cycle [13] via centrosome maturation, mitotic entry, centrosome separation, bipolar spindle assembly, chromosome alignment, cytokinesis and mitotic exit [14]. Dysfunctional AURKA regulation leads to genetic instability, potentially contributing to various malignant epithelial tumors, including head and neck, colon, ovarian, bladder, pancreatic and breast cancers [3, 15–19]. However, the effects of AURKA on LSCC metastasis and recurrence remain unknown. We hypothesized that AURKA promotes metastasis via the transformation of dormant cells from G0 phase to active division.

In the present study, we established a dormant LSCC cell model (D-Hep2 cells) to examine AURKA expression. We regulated AURKA expression in tumor Hep2 (T-Hep2) and D-Hep2 cells to assess dormant tumor cell revival. We also inhibited members of the FAK/PI3K/Akt pathway and found that AURKA might reverse tumor cell dormancy and contribute to LSCC metastasis and recurrence via FAK/PI3K/Akt pathway activation.

## RESULTS

### Low serum culture downregulates AURKA and induces Hep2 cell dormancy

**Figure 1:**
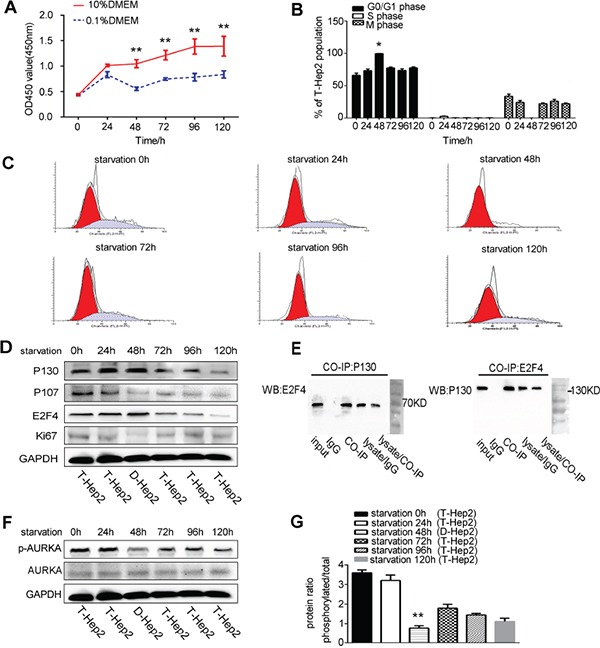
Culturing T-Hep2 cells with 0.1% FBS for 48 h induced dormancy and reduced AURKA expression **A.** T-Hep2 cell proliferation as measured by CCK8 assay at 0, 24, 48, 72, 96 and 120 h, T-Hep2 cells with 0.1% FBS showed stagnant growth at 48 h (**P<0.01). **B.** T-Hep2 cell population (%) after starvation (*P<0.05). **C.** T-Hep2 cell cycle as analyzed by flow cytometry T-Hep2 cells in 0.1% FBS were nearly stagnant in G0/G1 phase. **D.** Dormancy-related proteins were analyzed by western blotting P130 and E2F4 levels were high and P107 and Ki67 levels were low in serum-starved T-Hep2 cells. **E.** Co-IP was used to examine the E2F4-P130 complex **F.** p-AURKA and total AURKA in D-Hep2 cells and T-Hep2 cells as analyzed by western blotting, p-AURKA expression was low in D-Hep2 cells. **G.** Protein ratio in D-Hep2 and T-Hep2 cells (**P<0.01).

Serum starvation can induce cell dormancy [20]. T-Hep2 cells were cultured in DMEM with 0.1% or 10% FBS and CCK-8 assays were performed to assess proliferation at 0, 24, 48, 72, 96 and 120 h. T-Hep2 cells cultured with 10% FBS exhibited logarithmic growth, while cells cultured with 0.1% FBS were stagnant at 48 h (*P*<0.01, Figure 1A). Flow cytometry assay results showed that T-Hep2 cells cultured with 0.1% FBS for 48 h were in mainly G0/G1 phase (*P*<0.05, Figure 1B–1C). The dormancy-related proteins, P130, P107, E2F4 and Ki67, were detected by western blotting. P130 and E2F4 levels were elevated and P107 and Ki67 levels were decreased after culturing in 0.1% serum for 48 h (Figure 1D). Co-IP assay demonstrated the E2F4-P130 complex, unique in quiescent cells, in T-Hep2 cells cultured with 0.1% FBS for 48 h (Figure 1E). These results suggested that culturing T-Hep2 cells with 0.1% FBS for 48 h induced dormancy. AURKA was downregulated in dormant (D-Hep2) cells as compared with non-dormant (T-Hep2) cells as shown by western blotting (*P*<0.01, Figure 1F–1G), implying that AURKA is associated with LSCC cell dormancy.

### AURKA downregulation induces dormancy in T-Hep2 cells

**Figure 2:**
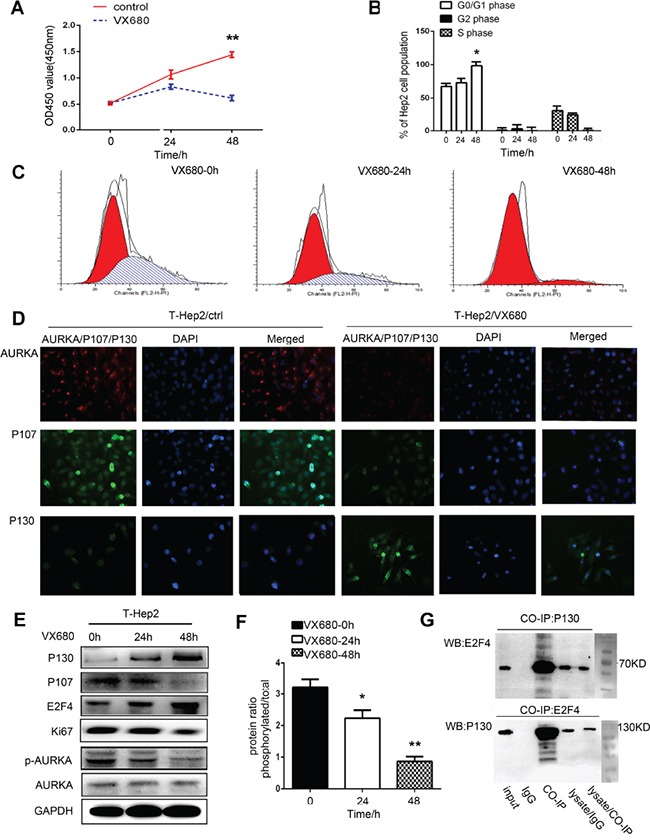
AURKA downregulation induced dormancy in T-Hep2 cells **A.** Effects of VX680 on T-Hep2 cell proliferation as measured by CCK8 assay at 0, 24 and 48 h Treated T-Hep2 cells exhibited reduced proliferation (**P<0.01). **B.** T-Hep2 cell population (%) after VX680 treatment (*P<0.05). **C.** Effects of VX680 on T-Hep2 cell cycle were analyzed by flow cytometry, T-Hep2 cells were arrested in G0/G1 phase 48 h after VX680 treatment. **D.** AURKA IF staining in T-Hep2 cells with or without AURKA inhibitor. AURKA and P107 levels were reduced, while P130 expression was higher in treated cells. **E.** Effects of VX680 on dormancy-related protein levels as analyzed by western blotting P130 and E2F4 levels were increased while p-AURKA, P107 and Ki67 were decreased in treated cells at 48 h. **F.** Protein ratio of p-AURKA in T-Hep2 cells (*P<0.05, **P<0.01). **G.** Co-IP was used to examine the E2F4-P130 complex in treated cells at 48 h.

T-Hep2 cells treated with the AURKA inhibitor, VX680 (100 nm/ml) [21], for 48 h showed reduced cell proliferation compared with the control group (*P*<0.01, Figure 2A). Flow cytometry assay results revealed that treated cells were arrested in G0/G1 phase (*P*<0.05, Figure 2B–2C). Immunofluorescence (IF) staining showed that AURKA was located in cytoplasm and P107 and P130 were located in nucleus. AURKA and P107 levels were reduced, while P130 levels were increased in T-Hep2 cells treated with VX680 compared with controls (Figure 2D). Western blotting showed that p-AURKA expression was decreased almost threefold. The dormancy-related proteins, P130 and E2F4, were upregulated and P107 and Ki67 were downregulated in VX680-treated cells (*P*<0.05, *P*<0.01, Figure 2E–2F). Co-IP showed that the E2F4-P130 complex existed in treated cells (Figure 2G). We concluded that AURKA inhibition could induce cell dormancy.

### AURKA overexpression reverses dormancy in D-Hep2 cells and enhances proliferation, migration and invasion

**Figure 3:**
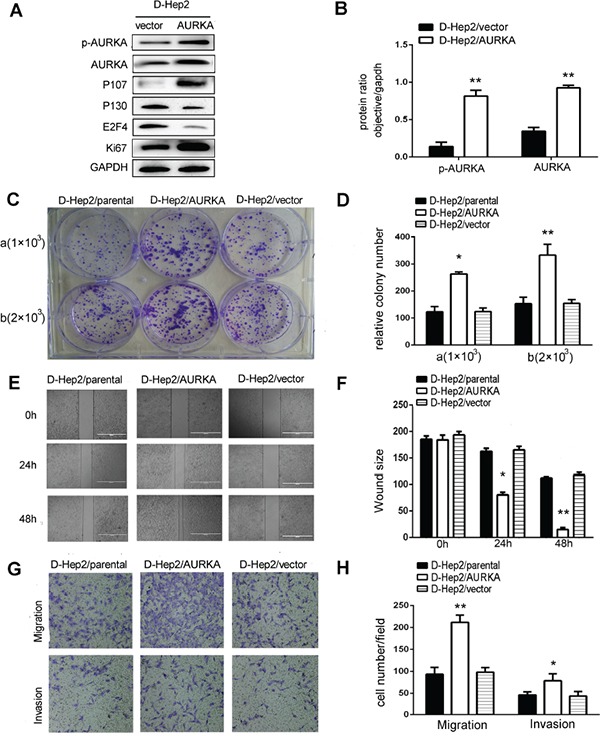
Migration, invasion and dormancy-related protein expression in D-Hep2 cells overexpressing AURKA **A.** Dormancy-related proteins were analyzed by western blotting, p-AURKA, AURKA, P107 and Ki67 levels were increased and P130 and E2F4 were reduced. **B.** Protein ratio in D-Hep2 cells. **C.** D-Hep2 cell proliferation was analyzed via colony formation assay, D-Hep2/AURKA cells enhanced cellular proliferation compared with D-Hep2/vector cells and D-Hep2/parental cells (**C/a**) plated cell number was 1×10^3^, *P<0.05; (**C/b**) plated cell number was 2×10^3^, *P<0.01). **D.** Relative D-Hep2 cell colony numbers. **E.** D-Hep2 cell mobility was analyzed via wound-healing assay. **F.** D-Hep2 cells/AURKA almost reached the middle of the scratch at 48 h (*P<0.05, **P<0.01). **G.** Cell migration and invasion were analyzed by transwell assays. **H.** AURKA promoted cellular migration and invasion (*P<0.05, **P<0.01).

We upregulated AURKA in D-Hep2 cells via plasmid transfection. Transfection was verified by western blotting. p-AURKA and AURKA levels were increased and P130 and E2F4 levels were reduced. P107 and Ki67 levels were increased in transfected D-Hep2 cells (D-Hep2/AURKA cells) compared with D-Hep2 cells transfected with vector only (D-Hep2/vector cells) (*P*<0.01, Figure 3A–3B). Colony formation assays showed that proliferation was enhanced in D-Hep2/AURKA cells (a:264±4.09; b:333±23.15) compared with D-Hep2/vector cells (a:124±7.77; b:155±8.09) and untreated cells (D-Hep2/parental cells) (a:123±11.39; b:153±14.25; *P*<0.05, Figure 3C–3D). In wound-healing assays, D-Hep2/AURKA cells were more motile at 48 h compared with D-Hep2/vector cells and D-Hep2/parental cells, indicating that AURKA promoted cell mobility (*P*<0.05, Figure 3E–3F). Similarly, more D-Hep2/AURKA cells migrated through transwell chambers (212±9.73) compared with D-Hep2/vector cells (98±6.00) and D-Hep2/parental cells (94±8.95). Finally, invasion assays indicated that D-Hep2/AURKA cells (79±9.24) moved through matrigel more frequently than D-Hep2/vector cells (44±6.01) and D-Hep2/parental cells (46±4.06) (*P*<0.05, Figure 3G–3H). These results suggest that AURKA overexpression could revive D-Hep2 cells and enhance cellular proliferation, migration and invasion.

### AURKA promotes D-Hep2 cell metastasis in nude mice

**Figure 4:**
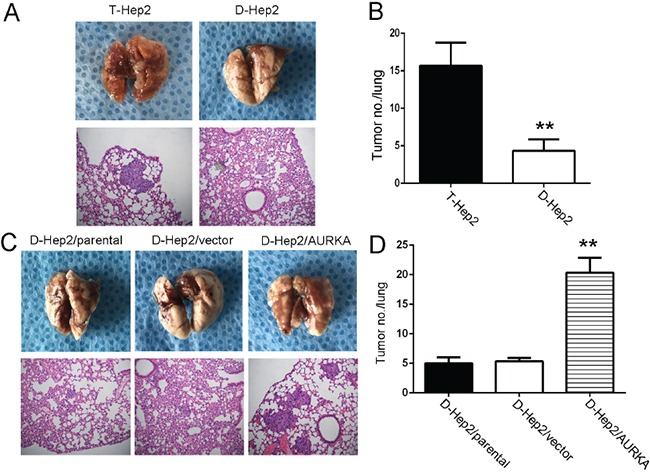
AURKA promotes D-Hep2 cell tumor metastasis in nude mice **A.** T-Hep2 cells and D-Hep2 cells were inoculated into nude mice and pulmonary nodules were observed after 45 days (*N*=5/group). H&E stains of pulmonary nodules (100×). **B.** Pulmonary tissue and nodules were quantified by H&E staining from T-Hep2, D-Hep2 (**P<0.01). **C.** D-Hep2/parental cells, D-Hep2/vector cells and D-Hep2/AURKA cells were inoculated into nude mice and pulmonary nodules were observed after 45 days (*N*=5/group). H&E stains of pulmonary nodules (100×). **D.** Pulmonary tissue and nodules were quantified by H&E staining from D-Hep2/parental cells, D-Hep2/vector cells and D-Hep2/AURKA cells (**P<0.01).

We previously reported that AURKA could enhance tumorigenesis *in vivo* [22]. T-Hep2, D-Hep2, D-Hep2/parental, D-Hep2/vector and D-Hep2/AURKA cellls were inoculated into nude mice by tail vain injection. Six weeks after inoculation, T-Hep2 cells (16±3.05) with higer AURKA expression demonstrated larger and more frequent lung metastases as compared to D-Hep2 cells (4±1.53) with lower AURKA expression (*P*<0.01, Figure 4A–4B). We also found higer lung metastasis risk in D-Hep2/AURKA cells (20±2.52) as compared to D-Hep2/parental (5±1.00) and D-Hep2/vector (5±0.58) (*P*<0.01, Figure 4C–4D). These results suggested that AURKA could revive dormant tumor cells to promote metastasis *in vivo*.

### AURKA revives dormant cells via FAK/PI3K/Akt pathway activation

**Figure 5:**
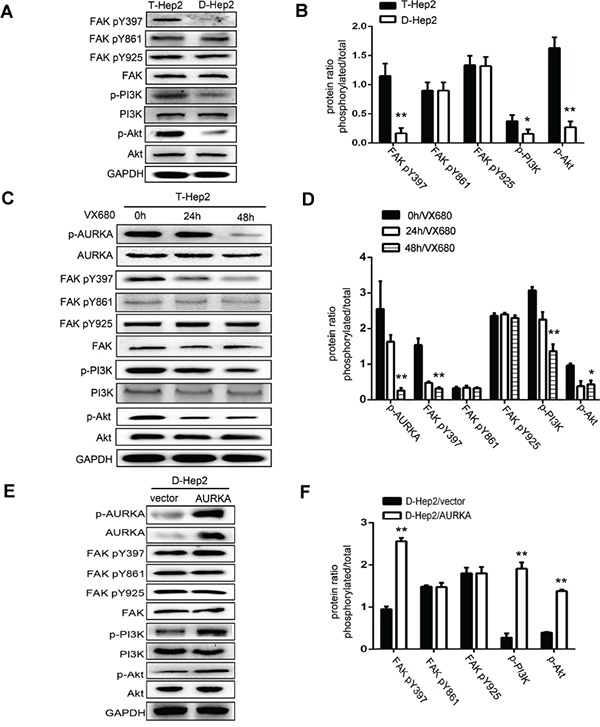
Effects of AURKA on FAK, PI3K, Akt activity **A.** Expression of FAK/PI3K/Akt pathway regulatory factors in T-Hep2 and D-Hep2 cells as analyzed by western blotting. These factors were overexpressed in T-Hep2 cells compared with D-Hep2 cells. **B.** Protein ratio in T-Hep2 and D-Hep2 cells (*P<0.05, **P<0.01). **C.** Effects of VX680 in T-Hep2 cells at 0, 24 and 48 h on the FAK/PI3K/Akt pathway as analyzed by western blotting. p-FAK (Tyr397), p-PI3K and p-Akt levels in treated T-Hep2 cells were lower than in untreated cells. **D.** Protein ratio in T-Hep2 cells (**P<0.01). **E.** Effects of AURKA upregulation in D-Hep2 cells on the FAK/PI3K/Akt pathway as analyzed by western blotting, p-FAK(Tyr397), p-PI3K and p-Akt levels were higher in transfected D-Hep2 cells compared to untransfected cells. **F.** Protein ratio in D-Hep2 cells (**P<0.01).

We hypothesized that AURKA might promote FAK/PI3K/Akt pathway activation to regulate LSCC tumor metastasis [23–29]. As measured by western blotting, levels of p-FAK (Tyr397), p-PI3K and p-Akt in T-Hep2 cells were higher than in D-Hep2 cells. Levels of p-FAK (Tyr861, Tyr925), FAK, PI3K and Akt were not altered (*P*<0.05, Figure 5A–5B). p-FAK (Tyr397), p-PI3K and p-Akt levels were higher in untreated T-Hep2 cells compared with cells treated with VX680, and in transfected (AURKA-overexpressing) D-Hep2 cells compared with untransfected cells. p-FAK (Tyr861, Tyr925), FAK, PI3K and Akt levels were consistently unchanged (*P*<0.05, Figure 5C–5F).

### FAK/PI3K/Akt pathway inhibition impacts dormancy-related proteins

**Figure 6:**
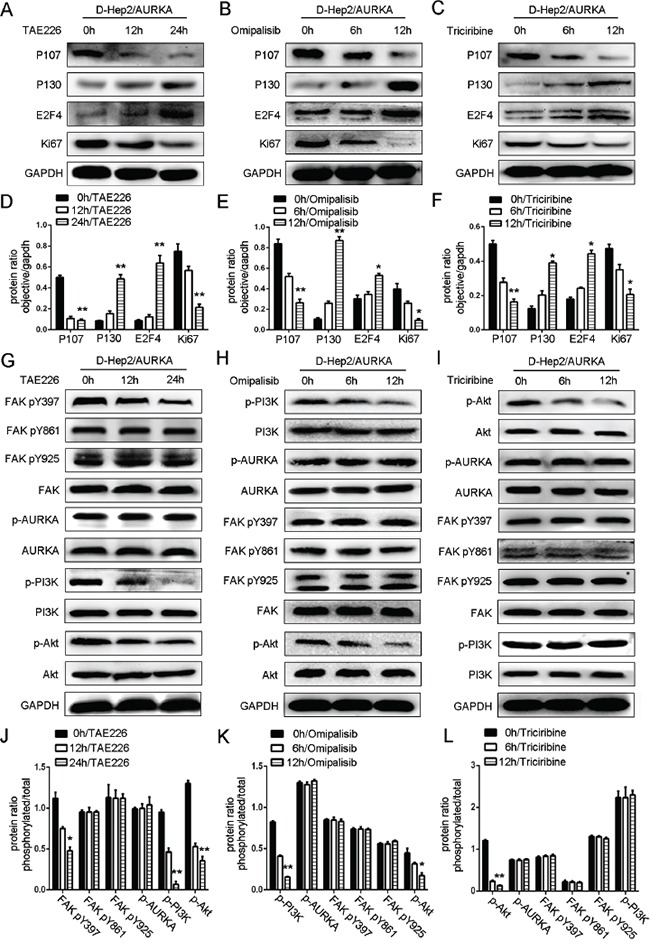
Effects of FAK/PI3K/Akt inhibition on dormancy-related protein expression Effects of FAK **A.**, PI3K **B.** or Akt **C.** inhibition in D-Hep2/AURKA cells on dormancy-related protein expression as analyzed by western blotting. P130 and E2F4 levels were increased, while P107 and Ki67 were decreased. Dormancy-related protein ratio in D-Hep2/AURKA cells treated with TAE226 **D.**, Omipalisib **E.** or Triciribine **F.** Effects of FAK **G.** inhibition in D-Hep2/AURKA cells on the FAK/PI3K/Akt pathway as analyzed by western blotting, p-FAK (Tyr397), p-PI3K and p-Akt levels were decreased and p-FAK (Tyr861, Tyr925), FAK, p-AURKA, AURKA, PI3K and Akt were not changed. Effects of PI3K inhibition **H.** in D-Hep2/AURKA cells on the FAK/PI3K/Akt pathway as analyzed by western blotting, p-PI3K and p-Akt levels were decreased and p-FAK (Tyr397, Tyr861, Tyr925), FAK, p-AURKA, AURKA, PI3K and Akt were not changed. Effects of Akt inhibition **I.** in D-Hep2/AURKA cells on the FAK/PI3K/Akt pathway as analyzed by western blotting, p-Akt levels were decreased, but p-AURKA, AURKA, p-FAK (Tyr397, Tyr861, Tyr925), FAK, p-PI3K, PI3K and Akt were not changed. Protein ratio in D-Hep2/AURKA cells treated with TAE226 **J.**, Omipalisib **K.** or Triciribine **L.** (*P<0.05, **P<0.01).

We treated cells with a FAK inhibitor (TAE226, 2.1 uM/ml) [30], a PI3K inhibitor (Omipalisib, 500 nM/ml) [31] or an Akt inhibitor (Triciribine, 5 uM/ml) [32] (dissolved in Dimethyl Sulfoxide (DMSO)) and assessed the impacts on the dormancy-related proteins, P107, P130, E2F4 and Ki67 by western blotting. In a time-dependent manner, TAE226 increased levels of P130 and E2F4 and decreased P107 and Ki67, indicating that FAK inhibition induced dormancy-like behavior (*P*<0.01, Figure 6A & 6D). p-FAK (Tyr397) expression was reduced almost twofold after 24 h TAE226 treatment, and p-PI3K and p-Akt were also decreased. While p-AURKA, AURKA, PI3K, Akt, p-FAK (Tyr861, Tyr925) and FAK were not changed, indicating that FAK is downstream of AURKA (*P*<0.05, Figure 6G & 6J).

In D-Hep2/AURKA cells treated with Omipalisib, we observed that P130 and E2F4 levels were increased, while P107 and Ki67 were decreased, demonstrating that PI3K inhibition induced dormancy-like behavior (*P*<0.05, *P*<0.01, Figure 6B & 6E). p-AURKA, AURKA, p-FAK (Tyr397, Tyr861, Tyr925), FAK, p-PI3K, PI3K, p-Akt and Akt expression was examined after treatment with Omipalisib for 0, 6 and 12 h. p-PI3K and p-Akt were decreased at 12 h, while p-AURKA, AURKA, p-FAK (Tyr397, Tyr861, Tyr925), FAK, PI3K and Akt were not altered, demonstrating that PI3K is downstream of AURKA/FAK (*P*<0.05, *P*<0.01, Figure 6H & 6K).

After treatment of D-Hep2/AURKA cells with Triciribine for 12 h, P130 and E2F4 levels were increased, while P107 and Ki67 were decreased, suggesting that Akt inhibition also induced dormancy-like behavior (*P*<0.05, *P*<0.01, Figure 6C & 6F). p-AURKA, AURKA, p-FAK (Tyr397, Tyr861, Tyr925), FAK, p-PI3K, PI3K, p-Akt and Akt levels were then examined after Triciribine treatment for 0, 6 and 12 h. p-Akt expression was decreased, while p-AURKA, AURKA, p-FAK (Tyr397, Tyr861, Tyr925), FAK, p-PI3K, PI3K and Akt levels were not, demonstrating that Akt was downstream of AURKA/FAK/PI3K (*P*<0.01, Figure 6I & 6L).

### FAK/PI3K/Akt pathway inhibition reduces D-Hep2/AURKA cell mobility, migration and invasion

**Figure 7:**
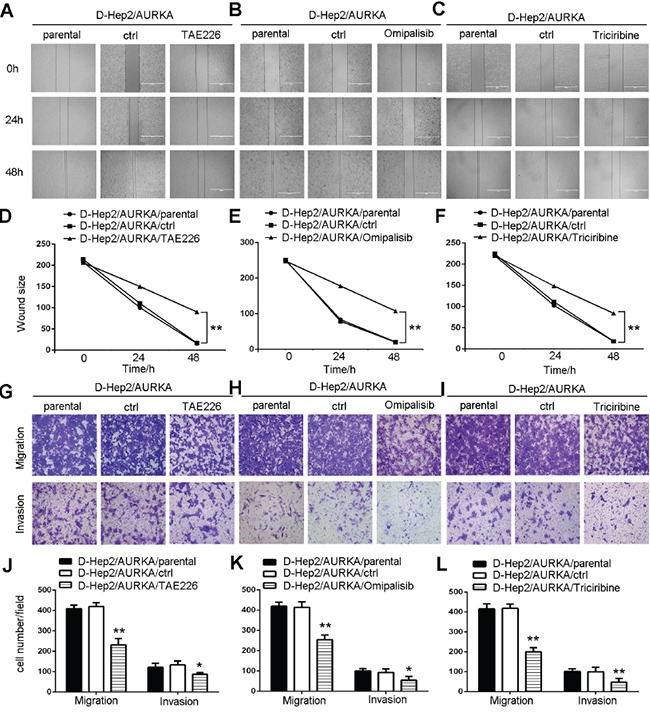
FAK/PI3K/Akt inhibition reduces D-Hep2/AURKA cell mobility, migration and invasion Effects of FAK **A.**, PI3K **B.** or Akt **C.** inhibition in D-Hep2/AURKA cells on cell mobility as assessed via wound healing assay. FAK, PI3K and Akt all promoted D-Hep2/AURKA cell mobility. D-Hep2/AURKA cell wound size with FAK **D.**, PI3K **E.** or Akt **F.** Effects of FAK **G.**, PI3K **H.** or Akt **I.** inhibition in D-Hep2/AURKA cells on cell migration and invasion as measured via transwell assay. FAK, PI3K and Akt each stimulated cell migration and invasion. Cell number in every field following FAK **J.**, PI3K **K.** or Akt **L.** inhibition. (*P<0.05, **P<0.01).

We applied wound-healing assays to test cell motility following treatment with inhibitors. Scratched wounds were observed for 0, 24 and 48 h after treatment. Results showed that D-Hep2/AURKA cells treated with TAE226, Omipalisib or Triciribine (D-Hep2/AURKA/TAE226 cells, D-Hep2/AURKA/Omipalisib cells or D-Hep2/AURKA/Triciribine cells) moved only slightly, while control D-Hep2/AURKA cells treated with DMSO only (D-Hep2/AURKA/ctrl cells) and untreated cells (D-Hep2/AURKA/parental cells) moved nearly to the middle of the scratch, indicating that FAK, PI3K and Akt promoted D-Hep2/AURKA cell mobility (*P*<0.01, Figure 7A–7C & 7D–7F).

In transwell migration assays, D-Hep2/AURKA cells treated with TAE226 (231±30.47) migrated less than D-Hep2/AURKA/ctrl cells (419±18.50) or D-Hep2/AURKA/parental cells (408±17.47). Invasion assays showed similar results, with D-Hep2/AURKA/TAE226 cells (87±8.62) invading less than D-Hep2/AURKA/ctrl cells (133±19.42) and D-Hep2/AURKA/parental cells (121±19.03) (*P*<0.05, Figure 7G & 7J). Migration and invasion capabilities were also tested in cells treated with PI3K or Akt inhibitors. D-Hep2/AURKA cells treated with Omipalisib (254±23.51) migrated less than D-Hep2/AURKA/ctrl cells (413±27.00) and D-Hep2/AURKA/parental cells (419±19.14). D-Hep2/AURKA/Omipalisib cells (54±19.01) also invaded less than D-Hep2/AURKA/ctrl cells (92±17.61) and D-Hep2/AURKA/parental cells (100±11.50) (*P*<0.05, Figure 7H & 7K). D-Hep2/AURKA cells treated with Triciribine (199±21.03) migrated less than D-Hep2/AURKA/ctrl cells (415±24.79) and D-Hep2/AURKA/parental cells (419±21.00). Similarly, D-Hep2/AURKA cells treated with Triciribine (47±18.15) invaded less than D-Hep2/AURKA/ctrl cells (102±12.12) and D-Hep2/AURKA/parental cells (100±22.50) (*P*<0.01, Figure 7I & 7L). These results showed that the FAK/PI3K/Akt pathway stimulated D-Hep2/AURKA cell mobility, migration and invasion.

## DISCUSSION

Tumor metastasis accounts for the majority of cancer-related deaths worldwide and the revival of dormant tumor cells may be one of the mechanisms related to metastasis. However, the precise molecular and cellular regulators involved in this transition remain poorly understood [33]. To study the role of dormant cells in tumor metastasis, we established a dormant cell model. Several interventions have been shown to induce cell dormancy, including starvation [20], short-term chemotherapy [34] and radiation [35]. Wilkie, *et al.* stated that tumor-immune dynamics in the micro-environment could inform tumor dormancy [36].

In this study, we induced dormancy (D-Hep2 cells) by culturing T-Hep2 cells with 0.1% FBS. The dormancy-related P130 and E2F4 proteins are abundant in quiescent cells [37, 38], the E2F4-P130 complex is unique in quiescent cells [21, 39–42], and the P107 and Ki67 proteins are rare [43]. E2F4, an E2F transcription factor, mediates the expression of cell cycle proteins [44]. The P130 and P107 proteins have considerable sequence homology compared with Rb [45–47], and are regulated by G1 cyclin-dependent kinases [48]. Ki67 is a proliferation indicator [49] that determines the risk of distant tumor recurrence [50].

We verified that T-Hep2 cells cultured with 0.1% FBS for 48 h were indeed dormant using the CCK8 assay, which showed that T-Hep2 cells were stagnant. Flow cytometry indicated that T-Hep2 cells were arrested in G0/G1 phase. Western blotting implied that P130 and E2F4 levels were elevated and P107 and Ki67 levels were decreased. Finally, Co-IP showed that the E2F4-P130 complex existed in dormant Hep2 cells. All results illustrated that D-Hep2 cells were successfully established. Notably, T-Hep2 cells cultured for more than 48 h did not maintain dormancy.

We investigated tumor dormancy as it relates to LSCC recurrence. Aurora kinase A (AURKA), a member of the Aurora serine/threonine kinase family [51], occurs from late G2 and M phase, whereas resting cells have low or undetectable levels of this enzyme [52]. Based on our previous study, AURKA expression was elevated in human LSCC as compared to adjacent normal tissues, and was associated with regional lymph node metastasis and TNM stage [3]. AURKA promoted Hep2 cell migration and invasion and enhanced tumorigenesis *in vivo* [22]. Here, we observed that AURKA overexpression could revive dormant tumor cells to promote tumor metastasis. To our knowledge, this is the first report of a relationship between AURKA and LSCC cell dormancy. In our study, AURKA expression was low in D-Hep2 cells and dormancy-related proteins were impacted by alterations in AURKA expression. The E2F4-P130 complex was observed in T-Hep2 cells after 48 h treatment with VX680. Furthermore, D-Hep2 cells overexpressing AURKA exhibited enhanced cellular proliferation, migration and invasion. Together, these results demonstrated that AURKA could revive dormant Hep2 cells to stimulate malignant progression in LSCC.

AURKA reportedly interacts with proteins such as p53, BRCA1, Plk1 and PI3K. Bolos, *et al.* noted that FAK interacted with Src to activate PI3K followed by Akt to promote tumorigenicity and metastasis [53]. Yao, *et al.* revealed cross-talk between AURKA and the PI3K pathway during Akt activation [54]. We therefore studied the role of the FAK/PI3K/Akt pathway in dormant tumor cell revival, and the interactions between AURKA and this pathway in promoting LSCC metastasis. The FAK/PI3K/Akt pathway was activated in T-Hep2 compared with D-Hep2 cells and was altered depending on AURKA expression. FAK/PI3K/Akt pathway inhibition also altered levels of dormancy-related proteins, suggesting that this pathway might regulate dormancy-like behavior along with D-Hep2/AURKA cell mobility, migration and invasion. Deservedly, there may be other more tumor signal pathways involved in the process except FAK/PI3K/Akt which deserve us to discover further.

In addition, VX680, TAE226, Omipalisib and Triciribine, inhibitors of AURKA, FAK, PI3k and Akt, respectively, reduced LSCC cell mobility, migration and invasion and lead to tumor regression. Therefore, drugs targeting the AURKA/FAK/PI3k/Akt molecules could be tested as single agent or combination therapies. Drug doses and schedules should be guided by further pre-clinical trials and correlative studies should be performed to test drug pharmacodynamics.

In conclusion, we demonstrated that AURKA may revive dormant tumor cells via FAK/PI3K/Akt pathway activation, thereby promoting migration and invasion in laryngeal cancer. FAK/PI3K/Akt/AURKA inhibitors might serve as potential targets for clinical LSCC treatment.

## MATERIALS AND METHODS

### Ethical statement

This study was approved by the Human Research Ethics Committee of Ruijin Hospital, School of Medicine, Shanghai Jiaotong University. Animals were approved by the Experimental Animal Ethics Committee of Ruijin Hospital based on the Institutional Animal Care and Use Committee (IACUC) of Shanghai Jiaotong University.

### Cell cultures

Human LSCC Hep2 (T-Hep2) cells were preserved by the Shanghai Institutes for Biological Sciences, Chinese Academy of Sciences. Cells were cultured with Dulbecco's modified Eagle's medium (DMEM) (Gibco company, USA) containing 0.1% or 10% Fetal Bovine Serum (FBS) (Gibco) with 100 IU/ml penicillin and 100 IU/ml streptomycin at 37°C and 5% CO_2_ in a humidified incubator.

### CCK8 assay

2×10^3^ cells in 100 ul of DMEM were seeded into 96-well plates. 10 ul of CCK8, used to measure cell proliferation, was added to every well. Cells were incubated for 2 h and OD450 absorbance values were measured. In our study, T-Hep2 and D-Hep2 cell proliferation was measured at 0, 24, 48, 72, 96 and 120 h. Cells treated with an AURKA inhibitor (VX680) (Selleck Chemicals, Houston, TX, USA) were measured at 0, 24 and 48 h.

### Flow cytometry

Cells under starvation conditions or treated with VX680 were seeded into 6-well plates and cultured to 60–70% confluence. Cells were trypsinized, rinsed three times with cold phosphate buffered saline (PBS) and fixed with 100% cold alcohol at 4°C overnight. Cells were then stained with 300 μl PI/Rnase Staining Buffer (BD Pharmingen) in the dark at 37°C for 30 min. FACS Calibur (Becton Dickinson, USA) was used to analyze cell cycle stage. Modfit Software (Becton Dickinson, USA) was used to quantify the number of cells in G0/G1, S or G2/M phase.

### Western blotting

Cells cultured under starvation conditions or treated with VX680, a FAK inhibitor (TAE226) (Selleck Chemicals, Houston, TX, USA), a PI3K inhibitor (Omipalisib) (Selleck Chemicals, Houston, TX, USA) or an Akt inhibitor (Triciribine) (Selleck Chemicals, Houston, TX, USA) were lysed with RIPA buffer (Pierce, Rockford, USA) containing 1% protease inhibitor cocktail. Protein concentrations were measured with the BCA Protein Assay Kit (Pierce, Rockford, USA). Proteins (100 μg/sample) were separated by 10% or 12.5% sodium dodecyl sulfate polyacrylamide gel electrophoresis (SDS-PAGE) for 2 h and transferred onto PVDF membranes (Millipore, MA, USA). Membranes were blocked with 5% nonfat milk in 1×TBST (150 mM NaCl, 0.05% Tween 20, 10 mM Tris–HCl, pH 8.0) for 2 h and incubated with primary antibodies overnight at 4°C. Primary antibodies included anti-P130 (1:2000, Santa Cruz), anti-P107 (1:2000, Santa Cruz), anti-E2F4 (1:2000, Santa Cruz), anti-Ki67 (1:2000, Santa Cruz), anti-p-AURKA (1:2000, Cell Signaling Technology), anti-AURKA (1:2000, Cell Signaling Technology), anti-FAK (1:2000, Cell Signaling Technology), anti-p-FAK (Tyr925, 1:2000, Cell Signaling Technology), anti-p-FAK (Tyr397, 1:2000, Cell Signaling Technology), anti-p-FAK (Tyr861, 1:2000, Abcam), anti-PI3K (1:2000, Abcam), anti-p-PI3K (1:2000, Abcam), anti-Akt (1:2000, Cell Signaling Technology), anti-p-Akt (1:2000, Cell Signaling Technology) and GAPDH (1:5000, Abcam). Membranes were incubated with secondary antibody (1:5000, Cell Signaling Technology) for 2 h and rinsed three times with 1×TBST for 10 min each. Proteins were visualized with an enhanced chemiluminescence detection system (Amersham Bioscience, Piscataway, NJ, USA).

### Co-immunoprecipitation assay

Co-IP assay was performed following the manufacturer's instructions (Thermo Scientific). Briefly, 10–75 μg of affinity-purified antibodies were immobilized, with anti-IgG as the control, and stored at 4°C. Then, 200–400 μl IP Lysis was added to each well of 6-well plates. Lysates were centrifuged at 13000 g for 10 min to extract proteins. For 1 mg of lysate, 40 μl of Control Agarose Resin slurry was added to a spin column to pre-clear lysate. Harvested proteins were incubated overnight with immobilized antibodies at 4°C, then separated by SDS-PAGE and transferred onto PVDF membranes for western blotting analysis. Primary antibodies included anti-P130 (1:1000, Anti-rabbit IgG, Santa Cruz) and anti-E2F4 (1:1000, Anti-mouse IgG, Santa Cruz). Secondary antibodies included anti-rabbit IgG (1:1000, Santa Cruz) and anti-mouse IgG (1:1000, Santa Cruz). Co-IP samples included total protein (4 μl), anti-IgG lysate combined with target protein (control group, 20 μl), anti-P130/E2F4 lysate combined with target protein (experimental group, 20 μl), anti-IgG lysate supernatant combined with target protein (20 μl) and anti-P130/E2F4 lysate supernatant combined with target protein (20 μl).

### Immunofluorescent staining

5×10^4^ cells, with or without VX680 for 48 h, were seeded into millicell ez slides (Millipore, MA, USA) and fixed with 4% paraformaldehyde (PFA) for 30 min. Slides were rinsed three times with PBS, blocked with 5% BSA in PBS containing 0.05% Triton for 1 h at room temperature and incubated overnight with primary antibodies at 4°C. Slides were then rinsed three times with PBS and incubated with secondary antibodies for 1 h at room temperature in the dark. Nuclei were visualized with DAPI (1:1000, Beyotime) in PBS for 5 min in the dark. Slides were rinsed three times in PBS and analyzed by fluorescent microscopy (10x). Primary antibodies included anti-p-AURKA (1:100, Anti-rabbit IgG, Cell Signaling Technology), anti-P130 (1:100, Anti-rabbit IgG, Santa Cruz) and anti-P107 (1:100, Anti-rabbit IgG, Santa Cruz). Secondary antibodies included Alexa Fluor® 488 goat anti-rabbit IgG and Alexa Fluor® 555 goat anti-rabbit IgG (1:1000, Santa Cruz).

### Transfections

AURKA siRNA (pEX-3(pGCMV/MCS/Neo)) was purchased from Shanghai GenePharma Company. 3×10^5^ cells were seeded into 6-well plates and incubated overnight. Cells were transfected with siRNA by lipofectamine 2000 (Invitrogen) and selected with 1200 ug/ml G418. Selected clones were verified by western blotting and frozen.

### Plate colony formation assay

Cells, with or without transfection, were seeded into 6-well plates at 1×10^3^ and 2×10^3^ cells/well. Cells were cultured in DMEM with 10% FBS for 3 weeks, washed twice with PBS and stained with crystal violet for 30 min. Cell colonies were counted in every well.

### Wound healing assay

A total of 1×10^6^ cells/well, with or without transfection or inhibitors, were inoculated into 6-well plates. After overnight incubation, 20 μl pipette tips were used to scratch the cells. Then, floating cells were washed away in PBS three times. Cells were photographed under a high-powered microscope (2x) at 0, 24 and 48 h.

### Cell migration and invasion assays

2×10^5^ cells, with or without transfection or inhibitors, were seeded in 200 ul of serum-free DMEM into the upper chambers of transwells (Boyden transwell chambers, Corning, MA, USA), and 600 μl of DMEM with 10% FBS was added into the lower chambers. Cells were cultured for 24 h and filters were stained with crystal violet for 30 min at room temperature. Cells in five random fields were counted under a high-power objective (10x). For invasion assays, upper chamber membranes were coated in matrigel (Becton Dickinson Labware, Bedford, MA, USA).

### *In vivo* metastasis

2×10^6^ T-Hep2, D-Hep2, D-Hep2/parental, D-Hep2/vector or D-Hep2/AURKA cells in 200 μl PBS intravenously injected via tail vein into 4-week-old male nude mice, which were purchased from the Institute of Zoology, Chinese Academy of Sciences. Metastatic nodules were counted by H&E staining after 45 days.

### Statistical analysis

CCK-8 assay, plate colony formation assay, flow cytometry, wound healing assay, cell migration and invasion assay and western blotting data were analyzed with GraphPad Prism 6 software and displayed as means ± SD. Differences between groups were assessed using Student's t test. *P*<0.05 was considered statistically significant.
